# Comparison of microvascular decompression and percutaneous balloon compression efficacy in patients with V2 idiopathic trigeminal neuralgia

**DOI:** 10.3389/fneur.2024.1406602

**Published:** 2024-10-15

**Authors:** Ye Ji, Junwu Wang

**Affiliations:** Department of Neurosurgery, Shengjing Hospital of China Medical University, Shenyang, Liaoning, China

**Keywords:** trigeminal neuralgia, microvascular decompression, percutaneous balloon compression, idiopathic, therapeutic effectiveness

## Abstract

**Objective:**

This study aims to compare the efficacy and long-term prognosis of microvascular decompression (MVD) versus percutaneous balloon compression (PBC) in patients with idiopathic V2 (maxillary branch) trigeminal neuralgia.

**Methods:**

We retrospectively analyzed the clinical information and follow-up data of patients who underwent surgical treatment for V2 idiopathic trigeminal neuralgia from January 2020 to January 2023. A total of 58 patients were included in the MVD group and 99 in the PBC group. All surgeries were performed by two physicians at the same center, with follow-up conducted by a separate, trained researcher. We compared the initial versus 12-month postoperative pain relief rates (scored using the BNI), surgical complications, and described pain relief rates after long-term follow-up in both groups using Kaplan–Meier analysis.

**Results:**

The study included a total of 157 patients (MVD 58, PBC 99). The median age of patients in the MVD group was lower than that in the PBC group (58 [51–65] vs. 63 [58–69], *p* = 0.002). There was no significant difference between the two groups in terms of pain relief rates initially after surgery and at 12 months (*p* = 0.521, *p* = 0.713). However, the MVD group had significantly better outcomes regarding postoperative facial numbness (*p* < 0.0001), masticatory weakness (*p* = 0.0017), and other complications (*p* = 0.04). Kaplan–Meier analysis showed that MVD provided a longer duration of pain relief than PBC (*p* = 0.0323), with most recurrences in both groups occurring within 1–2 years after surgery.

**Conclusion:**

There were no significant differences in significant pain relief rates between the two groups initially after surgery and at 12 months. However, the MVD group showed a clear advantage over PBC regarding postoperative facial numbness, masticatory weakness, and other complications; moreover, Kaplan–Meier analysis revealed that MVD offers a longer duration of pain relief for patients.

## 1 Introduction

Trigeminal neuralgia is a pain syndrome caused by the trigeminal nerve in the facial region, with an unclear mechanism. The pain of trigeminal neuralgia is primarily characterized by recurrent episodes of intense, stabbing pain, and it affects more women than men, significantly impacting patients’ quality of life. The treatment of trigeminal neuralgia mainly includes pharmacological and surgical interventions. Carbamazepine and oxcarbazepine are the first-line medications for treating trigeminal neuralgia (1). However, due to drug resistance and intolerable side effects experienced by some patients, surgical treatment is often chosen as the final option.

Dandy first described the neurovascular contact theory, which has been widely accepted by scholars. Numerous studies (2–4) have shown that neurovascular contact is closely related to morphological changes in the trigeminal nerve and trigeminal neuralgia pain. Trigeminal neuralgia can be classified etiologically into classical, idiopathic, and secondary types. Classical trigeminal neuralgia involves neurovascular contact with morphological changes in the trigeminal nerve, whereas idiopathic trigeminal neuralgia refers to cases with neurovascular contact but without morphological changes in the trigeminal nerve or without neurovascular contact (5–7). For classical trigeminal neuralgia, MVD is widely considered the treatment of choice (1). However, the optimal treatment for idiopathic trigeminal neuralgia remains uncertain. Most scholars tend to prefer PBC treatment because imaging does not show significant trigeminal nerve compression, and PBC is a less invasive treatment option compared to MVD (8, 9). However, several studies comparing the effects of PBC and MVD on pain control in TN patients have concluded that patients who underwent MVD had longer pain-free periods and lower complication rates than those who underwent PBC (10–12). Unfortunately, to date, there have been few comparative analyses of MVD and PBC surgery treatments specifically for patients with idiopathic trigeminal neuralgia.

The maxillary branch of the trigeminal nerve (V2) is a sensory nerve that exits the cranial cavity through the foramen ovale and distributes to the maxillary teeth, gingiva, and mucous membranes of the nasal cavity, which is one of the common sites of trigeminal neuralgia. Moreover, the study of a single branch of the V2 helps to reduce heterogeneity of patient selection, which increases the reliability of the results of this study. Therefore, this study retrospectively analyzed patients with idiopathic trigeminal neuralgia in the maxillary branch (V2), comparing the initial and long-term efficacy of MVD and PBC to guide pain management in this patient population.

## 2 Objectives and methods

### 2.1 Patient characteristics

**Figure 1:**
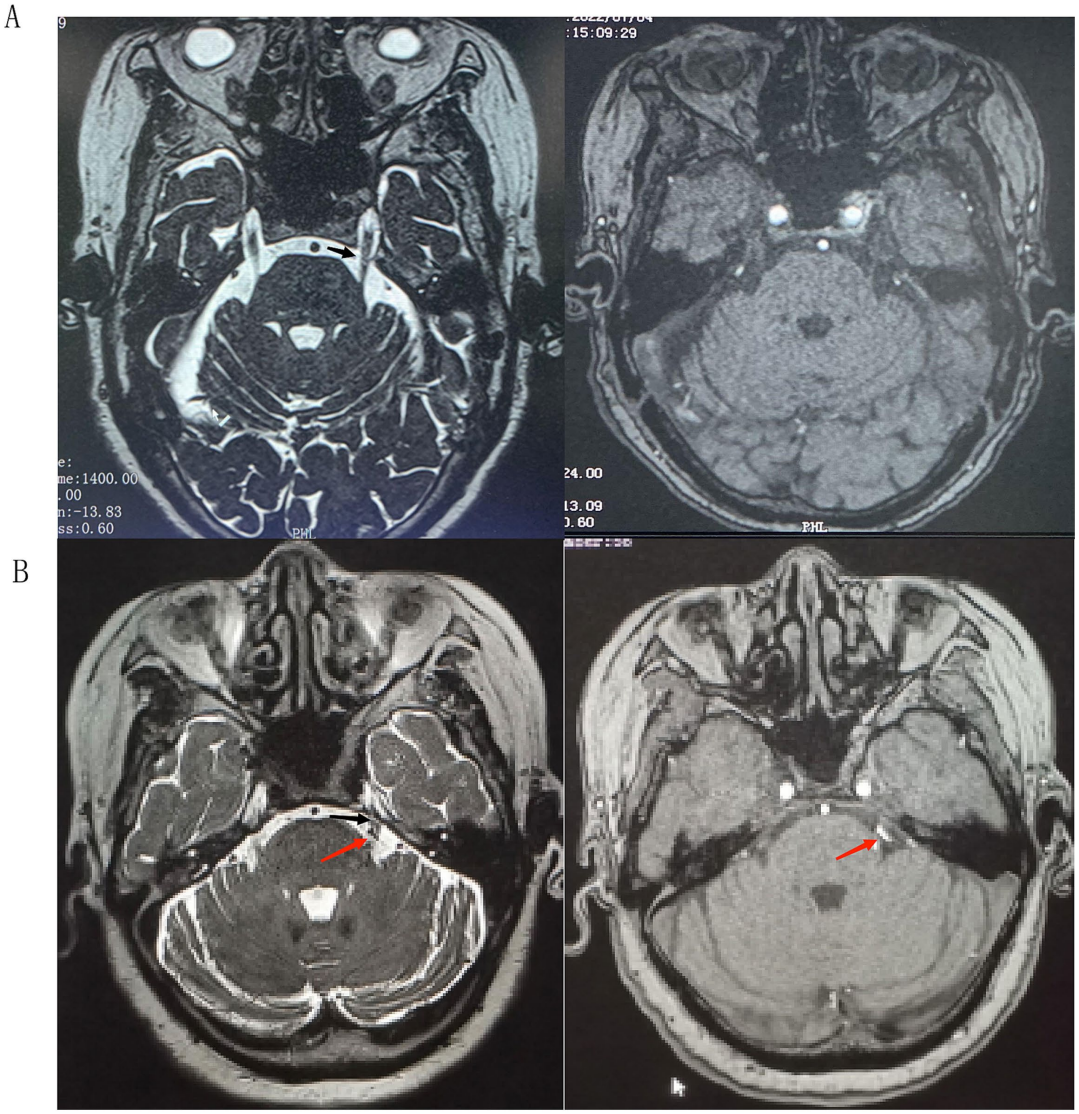
**(A)** 3D-SPACE and 3D-TOF-MRA sequences did not show obvious vascular nerve compression. **(B)** 3D-SPACE and 3D-TOF-MRA sequences showed obvious vascular compression, and the trigeminal nerve terminals appeared to be morphologically altered; black arrowheads: trigeminal nerves, red arrowheads: compression of blood vessels.

We systematically screened 424 patients with primary trigeminal neuralgia who underwent MVD or PBC treatment in the neurosurgery department of Sheng Jing Hospital from January 2020 to January 2023. The inclusion criteria were: (1) patients diagnosed with primary trigeminal neuralgia in the upper jaw branch (V2) according to the International Headache Society (IHS); (2) patients who underwent preoperative MRTA examination and met the criteria for idiopathic trigeminal neuralgia (with or without NVC but without morphological changes in the trigeminal nerve); (3) patients who received their first MVD or PBC treatment and had complete clinical and follow-up data (≥1 year). The exclusion criteria were: (1) patients with secondary, classic, or other branch-specific trigeminal neuralgia; (2) patients with multiple sclerosis; (3) patients with multi-organ dysfunction or tumors; (4) patients who discontinued treatment or interrupted follow-up. The choice of MVD or PBC treatment for a patient depends on the preoperative MRTA image (see Figure 1), the patient’s requirements and general condition, and the risks associated with the patient’s anesthesia and surgery. We collected demographic and clinical data, including age, sex, duration of TN at the time of surgery, painful site (left, right, or bilateral), presence of continuous pain, and effectiveness of sodium channel blockers.

### 2.2 Data collection and outcome measures

All patients underwent a minimum of 1 year of follow-up, with clinical outpatient follow-ups at 6 and 12 months postoperatively, and other follow-ups conducted through telephone questionnaires. Follow-up assessments were performed by trained independent researchers who were not involved in the treatment. The primary outcome measure was the Barrow Neurological Institute (BNI) pain score at 12 months postoperatively (13) (Table 1). Secondary outcome measures included the initial postoperative BNI pain score, occurrence of facial numbness, masseter weakness, and other complications. Complete pain relief was defined as a BNI score of I (no trigeminal pain without medication). Significant pain relief was defined as a BNI score of I or II. Pain recurrence was defined as a change from a good response with a BNI score of I or II to a lower level BNI score (III–V).

**Table 1:** Barrow Neurological Institute pain intensity scores.

Grade	Descriptions
I	Complete pain relief without any medication
II	Occasional pain, no medication required
III	Sometimes painful, fully controlled with medication
IV	Still have pain that is not fully controlled by medication
V	Pain persists, no relief

### 2.3 Surgical technique

#### 2.3.1 Microvascular decompression

The patient underwent general anesthesia and was positioned in a lateral decubitus position with the affected side facing upwards and the head secured in a headrest. A linear incision was made behind the ear, and a 3 cm × 3 cm bone flap was created below the occiput using a drill. The transverse sinus and sigmoid sinus junction were exposed above the bone flap. The cerebellar hemisphere was gently retracted inward and upward, and the entire length of the trigeminal nerve was visualized under a microscope. A thorough exploration of the nerve entry zone (NEZ) was performed in a 360° manner. If a typical artery loop compression was identified, it was separated using an appropriately sized Teflon pad. If no typical artery compression was found, but there was arterial contact without obvious nerve indentation or discoloration, or if only venous compression was identified, the blood vessel and nerve were isolated using a Teflon pad, and a high selective dissection of the trigeminal root at the NEZ was performed. Teflon pad isolation was also performed for potential vascular compression at the distal end of the trigeminal nerve. During the procedure, care was taken to protect the surface vessels of the brainstem and the large petrosal vein. After completing the subdural manipulation, the dura mater was sutured in place, the bone flap was repositioned and fixed, and the scalp was closed in layers.

#### 2.3.2 Percutaneous balloon compression

The patient received short-range general anesthesia and was positioned in a supine position. Hartel puncture technique was used with the puncture point located 3 cm outside the corner of the mouth. Under the guidance of a C-arm machine, a 14-gauge puncture needle with a blunt tip was slowly advanced to the entrance of the foramen ovale. The needle core was removed, and a 4F Fogarty catheter was introduced into the Meckel’s cave. The catheter tip was advanced 17 mm beyond the needle tip. The balloon was slowly inflated with iodinated contrast medium, and the shape and position of the balloon were observed. Approximately 0.5 mL of contrast medium was injected to inflate the balloon to a pear-shaped or teardrop shape to compress the trigeminal ganglion. Depending on the surgeon’s intraoperative judgment, the balloon was retained for approximately 3 to 5 min if the shape was unsatisfactory. If necessary, the needle was withdrawn and reinserted for balloon inflation and compression. After compression, the balloon was deflated, and the catheter and puncture needle were withdrawn. The patient’s cheek was compressed for several minutes.

### 2.4 Statistical analysis

Statistical analysis was performed using SPSS software version 26.0 (IBM Analytics, New York) and GraphPad Prism 8.0 for data visualization. A significance level of *p* < 0.05 was considered statistically significant for all tests. For categorical data, chi-square test or Fisher’s exact test was used for between-group comparisons. For continuous data, independent samples t-test or Wilcoxon rank-sum test was conducted. Postoperative facial pain prognosis was analyzed using Kaplan–Meier survival analysis, with the study endpoint defined as the occurrence of pain recurrence or the end of follow-up.

## 3 Results

**Figure 2:**
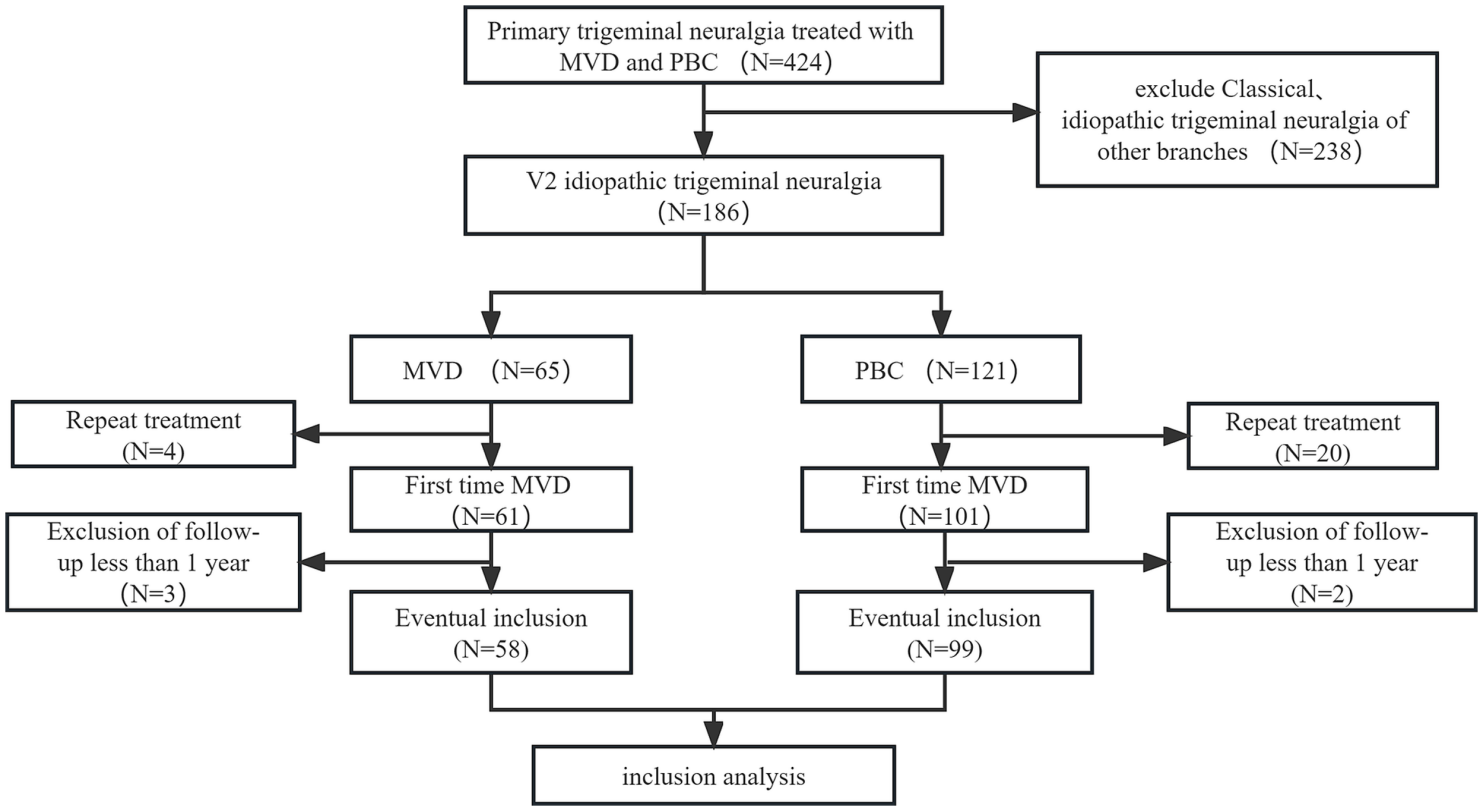
Patient screening flow chart.

A total of 267 patients were excluded from the study for various reasons: (1) classical or secondary trigeminal neuralgia (*n* = 238); (2) repeat treatment (*n* = 24); and (3) follow-up time less than 1 year (*n* = 5). Finally, 157 patients with V2 trigeminal neuralgia were included in the analysis, with 58 patients in the MVD group and 99 patients in the PBC group. The selection process is shown in Figure 2.

### 3.1 Baseline characteristics

In univariate analysis, the median age of patients in the MVD group was younger than that in the PBC group [58 (51–65) vs. 63 (58–69), *p* = 0.002]. The duration of TN was slightly shorter in the MVD group, but the difference between the two groups was not statistically significant [5 (4–5.5) vs. 5.5 (4.5–6), *p* = 0.059]. There was no significant difference between the two groups regarding gender, follow-up time, pain location, continuous pain, and response to sodium channel blockers (*p* > 0.05), as shown in Table 2.

**Table 2:** Baseline and procedure variables between MVD and PBC groups.

Baseline and procedure variables	MVD (*n* = 58)	PBC (*n* = 99)	*p-*value
Age, year, median (IQR)	58(51–65)	63(58–69)	0.002*
Female sex, *n*(%)	33(57%)	59(60%)	0.74
Duration of TN, year, median (IQR)	5(4–5.5)	5.5(4.5–6)	0.059
Follow-up time, month, mean ± SD	35.48 ± 2.82(18–36)	34.33 ± 4.56(16–36)	0.085
Site of disease			0.619
Right, *n*(%)	37(64%)	67(68%)	
Left, *n*(%)	21(36%)	32(32%)	
With persistent pain, *n*(%)	32(55%)	58(59%)	0.5
Sodium channel blockers are responsive, *n*(%)	30(52%)	59(60%)	0.337

MVD, microvascular decompression; PBC, percutaneous balloon compression; *p < 0.05.

### 3.2 Initial and 12-month pain relief rates after surgery

An analysis was conducted on the initial postoperative pain relief rates and the pain relief at 12 months for patients in the MVD and PBC groups. In the MVD group, after the initial postoperative BNI evaluation, 68.9% of patients experienced complete pain relief, and 89.6% of patients showed significant pain relief (BNI I + II). Six patients experienced some relief in pain compared to preoperative levels but did not reach significant relief (BNI III). In the PBC group, after the initial postoperative BNI evaluation, 73.7% of patients experienced complete pain relief, and 91.9% of patients showed significant pain relief (BNI I + II). Among the 8 patients who did not achieve significant relief, 1 patient showed no significant change in pain compared to preoperative levels (BNI IV), and 7 patients improved compared to the preoperative status (BNI III). There was no statistically significant difference between the two groups in terms of complete initial postoperative pain relief (*p* = 0.521) or significant pain relief rates (*p* = 0.631). At the 12-month outpatient follow-up, based on BNI scores, 87.9% of patients in the MVD group experienced significant pain relief (BNI I + II), while 85.9% of patients in the PBC group experienced significant pain relief (BNI I + II). There was no statistically significant difference between the two groups in terms of pain relief at the 12-month follow-up (*p* = 0.713), as shown in Table 3.

**Table 3:** Initial and 12-month postoperative pain relief rates for patients in MVD and PBC groups *n*(%).

BNI (grade)	Total (*n* = 157)	MVD (*n* = 58)	PBC (*n* = 99)
Initial postoperative pain relief			
I	113(72%)	40(68.9%)	73(73.7%)
II	30(19.1%)	12(20.7%)	18(18.2%)
III-V	14(8.9%)	6(10.3%)	8(8.1%)
12-month postoperative pain relief			
I	96(61.1%)	35(60.3%)	61(61.6%)
II	40(25.5%)	16(27.6%)	24(24.2%)
III-V	21(13.4%)	7(12.1%)	14(14.1%)

BNI, Barrow Neurological Institute; MVD, microvascular decompression; PBC, percutaneous balloon compression.

### 3.3 Complications

#### 3.3.1 Postoperative facial numbness and masseter weakness

**Figure 3:**
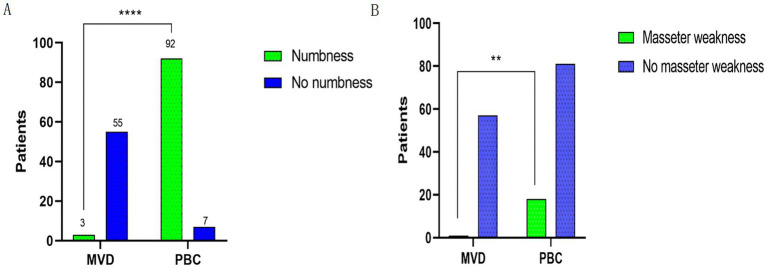
**(A)** Comparison of postoperative facial numbness between patients in the MVD and PBC groups. **(B)** Comparison of postoperative occlusal muscle weakness between patients in the MVD and PBC groups; *n* (%) were statistically analyzed. ****p < 0.0001, **p < 0.01.

An analysis was conducted on postoperative facial numbness and masseter weakness in the MVD and PBC groups, as shown in Figure 3. Regarding postoperative facial numbness, in the MVD group, 5.17% of patients experienced facial numbness, while 94.83% of patients did not have this symptom. In the PBC group, 92.93% of patients experienced facial numbness, while 7.07% of patients did not have this symptom. There was a significant statistical difference between the two groups (*p* < 0.0001). As for postoperative masseter weakness, in the MVD group, 1.72% of patients experienced symptoms of masseter weakness, while 98.28% of patients did not have this symptom. In the PBC group, 18.18% of patients experienced symptoms of masseter weakness, while 81.82% of patients did not have this symptom. There was a statistically significant difference between the two groups (*p* = 0.0017). During the follow-up period, we found that the symptoms of facial numbness and masseter weakness disappeared in the MVD group after 6 months postoperatively. In the PBC group, among the 81 patients with facial numbness, the symptoms disappeared after 6 months postoperatively, and in the 11 patients, the symptoms disappeared during the 12-month follow-up. Among the 18 patients with masseter weakness, the symptoms disappeared after 6 months of follow-up.

#### 3.3.2 Other complications

In the MVD group, 2 patients (3.4%) experienced facial numbness, and 2 patients (3.4%) developed herpes zoster. In the PBC group, 13 patients (13.1%) experienced facial numbness, 3 patients (3%) had diplopia, 5 patients (5%) developed herpes zoster, and 4 patients (4%) experienced hearing loss (Hearing can be roughly measured after awakening from post-operative anesthesia). The overall incidence of complications in the MVD group was 6.9%, while that in the PBC group was 25.3%. There was a statistically significant difference between the two groups (*p* = 0.04), as shown in Table 4. Patients with complications in both groups showed improvement during the 6-month follow-up period, which did not affect their quality of life.

**Table 4:** Comparison of remaining complication rates in MVD and PBC groups *n*(%).

	MVD (*n* = 58)	PBC (*n* = 99)	*p-*value
Complications			0.004
Dysesthesia	2(3.4%)	13(13.4%)	
Double vision	0	3(3%)	
Oral herpes	2(3.4%)	5(5%)	
Hearing impairment	0	4(4%)	
Intracranial infection	0	0	
Secondary cerebral infarction and hemorrhage	0	0	

MVD, microvascular decompression; PBC, percutaneous balloon compression.

### 3.4 Long-term pain relief rate after surgery

**Figure 4:**
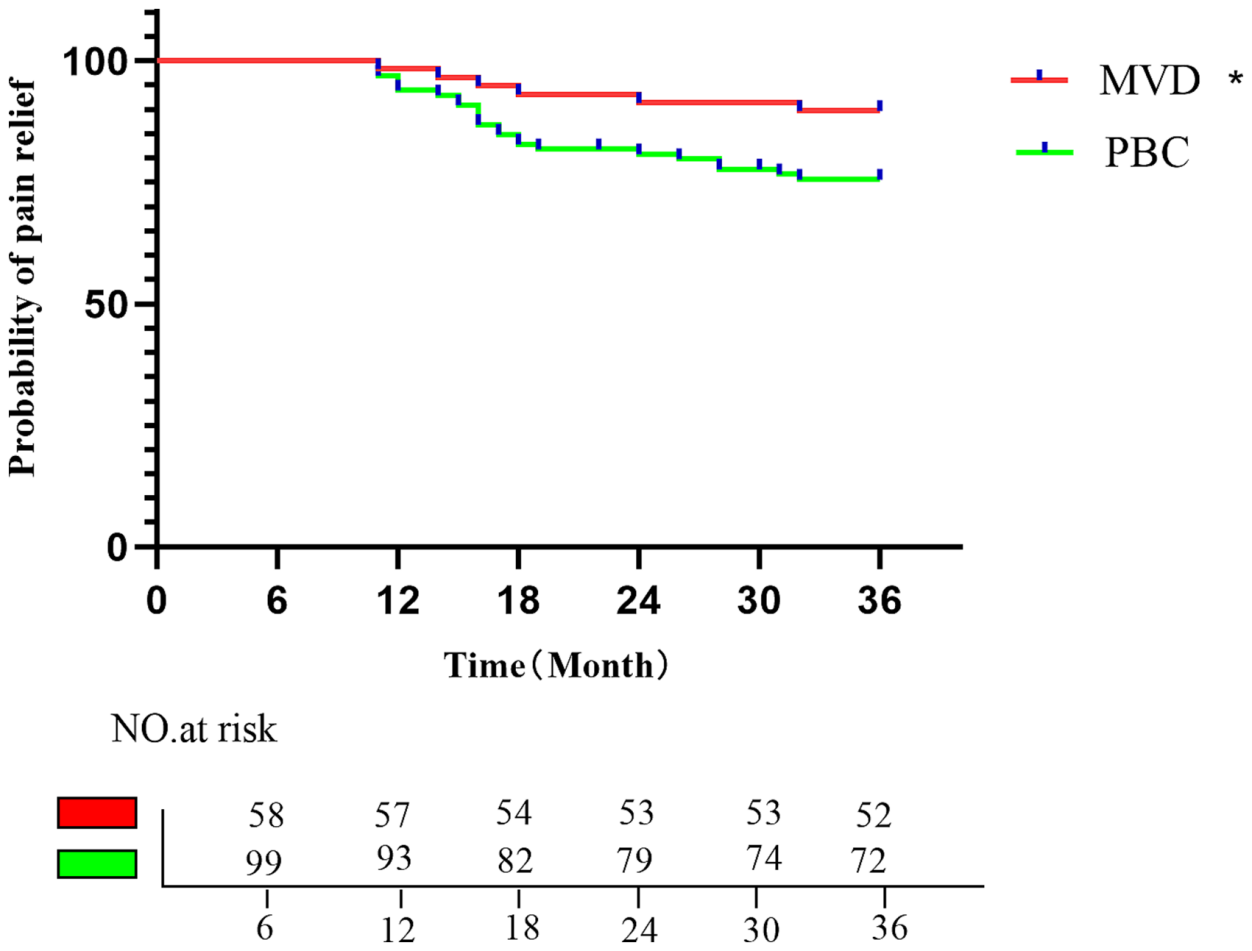
Kaplan–Meier analysis of the proportion of postoperative patients with pain relief in the MVD group and the PBC group; the rates of pain relief in the MVD group in the first, second, and third postoperative years were 96.55, 91.37, and 89.65%, respectively; the rates of pain relief in the PBC group in the first, second, and third postoperative years were 92.92, 80.79, and 75.6%, respectively. *p < 0.05.

A total of 157 patients who underwent MVD or PBC surgery were followed up for at least 1 year. The average follow-up time was 35.48 ± 2.82 months (range, 18–36 months) in the MVD group and 34.33 ± 4.56 months (range, 16–36 months) in the PBC group. After long-term follow-up, the significant pain relief rate decreased to 89.65% in the MVD group and 75.6% in the PBC group. Compared with the PBC group, the MVD group had a significantly longer duration of pain relief (*p* = 0.0323). Kaplan–Meier survival analysis was used to depict the time of pain recurrence after surgery in the MVD and PBC groups (as shown in Figure 4).

## 4 Discussion

Analytical studies comparing the efficacy between MVD and PBC for patients with solely idiopathic trigeminal neuralgia are scarce, making it necessary to conduct such research to provide appropriate treatment options for these patients. The primary reasons for exclusively choosing the V2 branch as the subject of this study are: (1) the V2 branch is a common site for TN; (2) aiming to reduce the heterogeneity of patient selection, thereby increasing the reliability of the study results. Ultimately, we found that there was no statistical difference in pain relief between the two groups in the initial postoperative period and at the 12-month follow-up. In terms of postoperative facial numbness, masticatory weakness, and other complications, the MVD group was significantly better than the PBC group; we used Kaplan–Meier analysis to examine the relationship between postoperative pain relief and follow-up time in the two groups, finding that patients in the MVD group had a longer duration of pain relief.

In recent years, there has been an increasing variety of surgical treatment options for trigeminal neuralgia. The most common treatment methods include microvascular decompression (MVD) and percutaneous procedures. MVD is the most classic and commonly used surgical approach for treating trigeminal neuralgia, and it is based on the vascular compression theory. Gardner (14) first performed vascular decompression surgery, followed by Jannetta (15) and Barker et al. (16), who introduced the use of microsurgical techniques and promoted MVD as the primary treatment for trigeminal neuralgia. Nowadays, the effectiveness of MVD treatment has been widely accepted by neurosurgeons, especially for patients with classic trigeminal neuralgia, where MVD is considered the preferred treatment method. Early studies indicated (17) that the postoperative pain relief rate for patients undergoing MVD ranged from 83 to 98%. A prospective study (18) involving the initial treatment with MVD found a pain relief rate of 83% at 1 year postoperatively and 61% at 5-year follow-up. Furthermore, another prospective study (19) reported that 88% of patients were pain-free at 24 months post-MVD follow-up.

However, there is a difference of opinion regarding whether MVD treatment has the same pain relief effect for patients who do not have vascular contact with the nerve or have contact but do not show significant morphological changes in the trigeminal nerve. A study (20) has indicated that compared to typical TN patients, MVD has a poorer pain relief effect for atypical TN patients. However, this study classified patients into typical and atypical based on symptoms rather than preoperative MRTA imaging, which may introduce bias into the results. In a prospective study (21) involving 115 trigeminal neuralgia patients who underwent MVD treatment, it was found that MVD was effective for idiopathic TN with vascular contact (without morphological changes in the trigeminal nerve), but the degree of effectiveness differed from that of classic TN. The patient inclusion in this study was based on preoperative MRTA imaging analysis, and patients with NVC accompanied by morphological changes in the trigeminal nerve were excluded. Key factors influencing the choice of surgical approach for patients are anesthesia tolerance and surgical risk assessment, which are to some extent dependent on patient age. In this study, the MVD group had a younger age profile compared to the PBC group. Regarding the initial postoperative pain relief rate (BNI I + II), the MVD group achieved a rate of 89.6%, and the pain relief rate at 1 year postoperatively was 87.9%, which is consistent with previous research on primary trigeminal neuralgia patients receiving MVD treatment.

For patients with idiopathic trigeminal neuralgia, most scholars tend to prefer percutaneous procedures as the treatment of choice (8, 9). The advantages of this technique include high efficacy, short hospital stay, low mortality rate, and the ability to be repeated multiple times (22), avoiding complications such as brain tissue hemorrhage, infarction, venous rupture, and cerebral edema that may arise from open MVD surgery, although such complications are rare with MVD itself. A study on the effectiveness of PBC in improving pain and quality of life in postoperative patients (23) showed an initial significant improvement rate of 83.8% after PBC. The pain relief rates at 1, 2, and 3 years postoperatively were approximately 94.2, 87.6, and 83.8% respectively, based on BNI I-II classification. Li et al. (24) study found that the initial pain relief rate after PBC could reach 98.11%, with a long-term pain relief effect of 96.23%. A meta-analysis comparing the efficacy of MVD and PBC in treating TN found that both MVD and PBC had high and similar pain relief rates, but MVD was associated with a lower recurrence rate (11).

In our study, for patients with idiopathic trigeminal neuralgia, we found an initial pain relief rate of 91.9% after PBC, and an 85.9% pain relief rate at 1 year postoperatively. Compared to the MVD group, the PBC group showed a higher trend in the initial pain relief rate after surgery, although there was no statistically significant difference between the two groups. However, in terms of the pain relief rate at 1 year postoperatively, the PBC group had a greater decline trend. We observed in our study’s follow-up that the main reason for this was the significantly higher number of recurrences within 1 year in the PBC group compared to the MVD group, leading to a decrease in the pain relief rate. Overall, there was no statistically significant difference between the PBC and MVD groups in terms of the initial pain relief rate or the pain relief rate at 12 months postoperatively.

Postoperative complications and long-term symptom relief rates are key indicators for comparing the safety and efficacy of different surgical procedures. In a previous study comparing Microvascular Decompression (MVD) and Percutaneous Balloon Compression (PBC) (25), no adverse events such as cerebrospinal fluid leakage, subdural hematoma, wound infection, or death were observed in either the MVD or PBC group. However, facial numbness was reported in 82.4% of patients in the PBC group, with 47.1% experiencing persistent numbness. In comparison, only 18.7% of patients in the MVD group had facial numbness. Another study (24) also found that the incidence of facial numbness and jaw muscle weakness was significantly higher in patients treated with PBC compared to those treated with MVD. Follow-up observations showed that these symptoms significantly diminished around 3 months postoperatively and nearly completely resolved after 6 months. Research (10, 26, 27) has indicated a correlation between sensory disturbances and pain relief, suggesting that more lasting pain relief can be achieved through destructive injuries. Additionally, postoperative numbness is an important predictive factor for favorable outcomes and long-term non-recurrence in percutaneous procedures.

In our study, we found that 92.93% of patients in the PBC group experienced facial numbness, 18.18% had jaw muscle weakness, and 25.3% developed other complications such as reduced sensation, herpes labialis, diplopia, and hearing impairment. In the MVD group, 5.17% of patients had facial numbness, 1.72% had jaw muscle weakness, and 6.9% experienced other complications like herpes labialis and sensory disturbances. No adverse events such as cerebral infarction, intracranial hemorrhage, intracranial infection, or death occurred in either group. The incidence of postoperative numbness in our study was higher than in previous research, which we attribute to variations in intraoperative balloon pressure maintenance. When the surgeon sensed a decrease in balloon pressure, 0.1–0.2 mL of iodized iohexol was continuously injected to maintain the pressure. Encouragingly, almost all patients with complications experienced complete symptom relief during the 6-month follow-up. In the long-term follow-up, we found that the MVD group had a longer pain-free period, and pain recurrence in both groups mainly occurred between 1 and 2 years postoperatively. Some reports have suggested a 3-year recurrence rate of 27.1% for MVD (28), but in our study, we did not observe such a high recurrence rate. Instead, the 3-year recurrence rate was 27.3% in the PBC group. Compared to the PBC group, MVD demonstrated a lower recurrence rate in the treatment of idiopathic trigeminal neuralgia.

This study also has certain limitations. Firstly, it is a retrospective study, so it cannot fully control for variables. Additionally, the follow-up period is too short to observe the recurrence of the condition in some patients. Thirdly, the lack of preoperative physical examination data and preoperative incidence of facial numbness limits a more robust predictive analysis. Finally, although we diagnosed patients as having idiopathic trigeminal neuralgia based on their MRTA images before the Microvascular Decompression (MVD) surgery, we still found that some patients exhibited clear vascular compression of the nerve during the procedure, which could introduce a certain degree of bias into the results.

## 5 Conclusion

There was no statistically significant difference in the initial and 12-month pain relief rates between Microvascular Decompression (MVD) and Percutaneous Balloon Compression (PBC) in patients with idiopathic trigeminal neuralgia involving the V2 branch. However, the MVD group showed significantly better outcomes in terms of postoperative facial numbness, masseter weakness, and other complications compared to the PBC group. Kaplan–Meier analysis revealed that the pain relief duration was longer in the MVD group. Therefore, in the long term, MVD appears to be more effective than PBC for the treatment of idiopathic trigeminal neuralgia. However, these conclusions still require validation through large-scale, multicenter prospective studies.

## Data Availability

The original contributions presented in the study are included in the article/supplementary material, further inquiries can be directed to the corresponding author.
